# Improving viral load testing using a quality improvement approach in Blantyre, Malawi

**DOI:** 10.1371/journal.pone.0269062

**Published:** 2022-06-08

**Authors:** Angella J. Kamwendo, Mina C. Hosseinipour, Juliana Kagura

**Affiliations:** 1 Department of Epidemiology and Biostatistics, School of Public Health, Faculty of Health Sciences, University of the Witwatersrand, Johannesburg, South Africa; 2 University of North Carolina Project, Lilongwe, Malawi; 3 Department of Medicine, University of North Carolina School of Medicine at Chapel Hill School of Medicine, Chapel Hill, NC, United States of America; Federal University of Sergipe, BRAZIL

## Abstract

**Background:**

Viral load (VL) testing coverage remains low particularly in resource limited countries despite recommendation by World Health Organization, and Malawi is no exception. A quality improvement (QI) approach was used to improve VL testing coverage from 27% to a target of 80% at an urban health facility in Malawi.

**Methods:**

A QI study employing a time-series quasi-experimental design with no comparison group was conducted at Chilomoni health centre in Blantyre from April 2020 to July 2020. A retrospective record review of all patient records (257) from 8 weeks before the study was conducted to determine baseline VL testing coverage. Root cause analysis of low VL testing coverage was done using fish-bone tool and factors prioritized using a Pareto-chart. Priority factors included inadequate capacity to update electronic medical records and competing tasks. Change ideas were identified and prioritized using an effort-impact matrix. Two change ideas; *re-orienting ART providers on VL test order in EMR* and *dedicated ART provider to serve VL tested patients* were implemented and tested in 5 Plan-Do-Study-Act (PDSA) cycles from the Model for Improvement (MFI), each lasting one week. The latter was tested, and adapted in 3 cycles, and eventually adopted for monitoring for another 5 weeks. VL testing coverage was tracked throughout the study using run charts and p-charts.

**Results:**

VL testing coverage increased from 27% to 71% by the end of the study, with children aged 0 to 14 years having the lowest coverage throughout the study.

**Conclusion:**

The MFI as a QI approach improved VL testing coverage through implementation of contextualized change ideas. A reliable data system, leadership buy-in and commitment are important for sustained improvement. Future research should focus on evaluating sustainability of improved VL testing coverage at the health facility and assessing barriers to VL testing among the paediatric population.

## Introduction

The World Health Organization (WHO) recommends viral load (VL) testing as means of monitoring the response of clients with HIV to Antiretroviral Therapy (ART) and the scale up of routine viral load testing in resource limited countries [1]. Viral load testing assists in early detection of ART failure, prevent unnecessary switches to other regimens thereby reducing ART drug resistance and enables health service providers to take early action in the management of HIV clients who have portrayed signs of ART failure [2]. This, in turn is important for maintaining and sustaining a suppressed viral load in individuals on ART thereby reducing transmission and realizing better patient outcomes [3]. A 90 percent reduction in new HIV infections and deaths from HIV related illnesses is the key to eliminating HIV/AIDS as a public health concern [4]. This impact can only be realized upon achieving suppressed viral loads among individuals on ART according to the HIV cascade, hence the need to capitalize on VL testing [5]. Regardless of the recommendation by WHO, VL testing coverage is still low in many parts of the world, with resource limited countries experiencing the most challenges [6].

In Malawi, VL testing for individuals on ART was launched in August 2012 and has been a priority of the Ministry of Health in the country [7]. However, Malawi faces challenges of low VL testing coverage (19%) despite having a policy in place and financial support for HIV services [6, 7]. In 2018, VL testing coverage ranged from 15% to 54% across 27 districts in Malawi, with only 2 districts having coverage above 40% [8]. This is below the 70% VL testing coverage target for 2018 set by the Ministry of Health in the country [7]. Low coverage on VL testing has implications on the Malawi government’s ability to maintain and sustain suppressed viral loads among individuals with HIV hence continued transmission and poor patient outcomes. General factors such as transportation and laboratory equipment, affecting VL testing coverage and common to resource limited countries have already been established [9, 10]. However, there exists a gap in contextual factors specific to a site or health facility that affect VL testing coverage, as well as simple methods for developing context specific and tailored solutions at these facilities using a QI framework. Such a gap can be covered using quality improvement (QI) as it allows for systematic changes and rapid tests of tailored interventions [11].

At Chilomoni health centre in Blantyre Malawi, substantial proportion of patients had missed VL tests that were due. In April 2020, VL testing coverage for pregnant and lactating women as well children was pegged at 27%. We therefore set out to improve VL testing coverage at the health facility using the Model for Improvement (MIF) framework. Specifically, we explored the factors affecting VL testing coverage at the health facility, identified change ideas and tracked the outcome measure throughout the study. In this paper, we describe the methods used to develop interventions and components of the interventions that brought about change in the VL testing coverage at the health facility.

## Materials and methods

### Study design

We conducted a QI study utilizing a time series (AB) quasi-experimental study design with no comparison group from April 2020 to July 2020 at Chilomoni Health Center ART clinic in Blantyre, Malawi. The study design was employed to allow continuous monitoring of the outcome at different time points, from baseline to the end of the study [12].

### Study site

Chilomoni health centre is government owned and recognized as an urban health facility. The ART clinic at the health centre was established in 2006 and serves 4400 individuals on ART. It is located at latitude 15°46’14.9" in the South and longitude -34°58’54.9" in the East in Blantyre city. Blantyre city is situated in the southern region of Malawi and has one of the highest HIV prevalence, 17.7% [13]. In addition, Blantyre has a VL monitoring coverage between 25 and 30%, and is among the lowest performing districts in the country [14].

### Study population

We initially planned to include patient records of all individuals who were HIV positive, had been on ART for 6 months or more, and were due for a VL test from 8 weeks prior to the study and during the study period. However, due to COVID-19, the Malawi Ministry of Health issued new HIV services guidelines which stipulated that routine VL testing for the general population was put on hold. The service was only available to pregnant and lactating women, children and those in need of targeted testing [15]. Therefore, the study only included pregnant and lactating women as well as children and excluded those in need of targeted testing as identification of this group requires extra attention and would require its own set of interventions. Their records were used to measure VL testing coverage at baseline and throughout the rest of the study.

The study also included health care providers who were key in the process of VL testing at the ART clinic in order to investigate factors affecting VL testing coverage and development of a change ideas. These health care providers included clinicians, nurses, Expert Clients, clerks and Health Surveillance Assistants (HSAs).

### Sample procedure

For the VL testing coverage component, all 428 records belonging to individuals (pregnant and lactating women and children) due for a routine VL test during the study, including the baseline period were included in the study. This was done in order to get precise VL testing coverage results, but also because of the shrinkage of the study population due to COVID-19 HIV service delivery guidelines.

On the other hand, purposive sampling was used to determine health care providers to be included in the study for investigation of factors affecting VL testing coverage at the health facility and development of change ideas. Only those health care providers who were directly involved in the VL testing process at the ART clinic were involved in the study. A total of 15 health care providers were used for this exercise. Purposive sampling was also used to determine the health facility where the study would take place; expert guidance was sought from the Blantyre District Health Office.

### The viral load testing process and measures

In this study, we sought to improve VL testing during ART clinic days. At Chilomoni Health Centre, the initial process of VL testing started with health talks at the waiting area and topics such as viral load testing were covered. At the waiting area, patients underwent VL test eligibility screening done by any available health care provider. From the waiting area, the patient went to the reception to collect their ART card and to get their vitals such as weight and height checked. The patient would then go back to the waiting area or the ART provider’s office for consultation and medication. If the patient was found eligible for a VL test at the waiting area or in the ART providers office, the patient was sent to the VL testing room for a sample to be drawn and then proceed with all other activities at the ART clinic. If the patient is a Mother-Infant pair (MIP), the pair would first go to the Exposed Infants Diagnosis (EID) desk for consultation and medication for the infant, after which the mother would go to the waiting area to proceed with all other processes.

To track improvement of the process, the outcome that was being tracked during the study was VL testing coverage. For purposes of this study, it was defined as the proportion of individuals who had been on ART for 6 months or more and were due for a VL test, whose sample for a VL test was collected, and their names had been documented in the VL testing register regardless of whether they received a result or not. The number of people in the VL register (numerator) was compared to the number of people who have been on ART for 6 months or more and were due for a VL test (denominator) during the study period. The VL register was the only source of complete VL testing data, unlike Electronic Medical Records (EMR).

Apart from the outcome measure, we also collected data on process and balancing measures throughout the study (**Table 1**). This was done in order to ensure change ideas were being implemented as planned and consequences of implementing the change ideas on other parts of the system were being tracked. Data on process and balancing measures were collected and reviewed on weekly basis with the testing of each change idea. The outcome was measured throughout the study.

**Table 1:** Change ideas, process and balancing measures.

Change Idea	Process Measures	Balancing Measures
**Re-orienting ART providers on VL test order in EMR**	Proportion of ART providers oriented on ART clinic day	Proportion of VL tests ordered in EMR but patient did not receive a VL test
	Proportion of VL tests ordered in EMR	
**Dedicated ART provider to serve VL test patients i.e. ordering VL tests in EMR**	Proportion of patients who were eligible for a VL test and had their health passport held at the reception, received a VL test.	Proportion of VL tests ordered in EMR but patient did not receive a VL test
	Proportion of VL tests ordered in EMR	

### Data collection

Data collection during the study was conducted in 2 phases:

#### Phase I

This phase comprised of the following steps:

*Step 1*: *Baseline assessment*. Since the health facility already had a QI team, 3 QI team representatives were assigned by the health facility In-charge to work in this project: a Data Clerk, VL focal person and nurse. During the baseline assessment, a retrospective record review of pregnant and lactating women as well as children who were due for a VL test from 17^th^ February 2020 to 11^th^ April 2020 (8 weeks) was conducted. Records were taken from the ART attendance register and from an electronic medical record (EMR) system. These records were compared to records in the VL register and/or the EMR to check if individuals who were due had a sample collected for a VL test. Data from records was extracted by the investigator and data clerk into Microsoft Excel. Overall baseline VL coverage at the health facility was 27%; among women and lactating women 34% and 11% among children. The target was to get to 80%, motivated by the national target.

**Fig 1:**
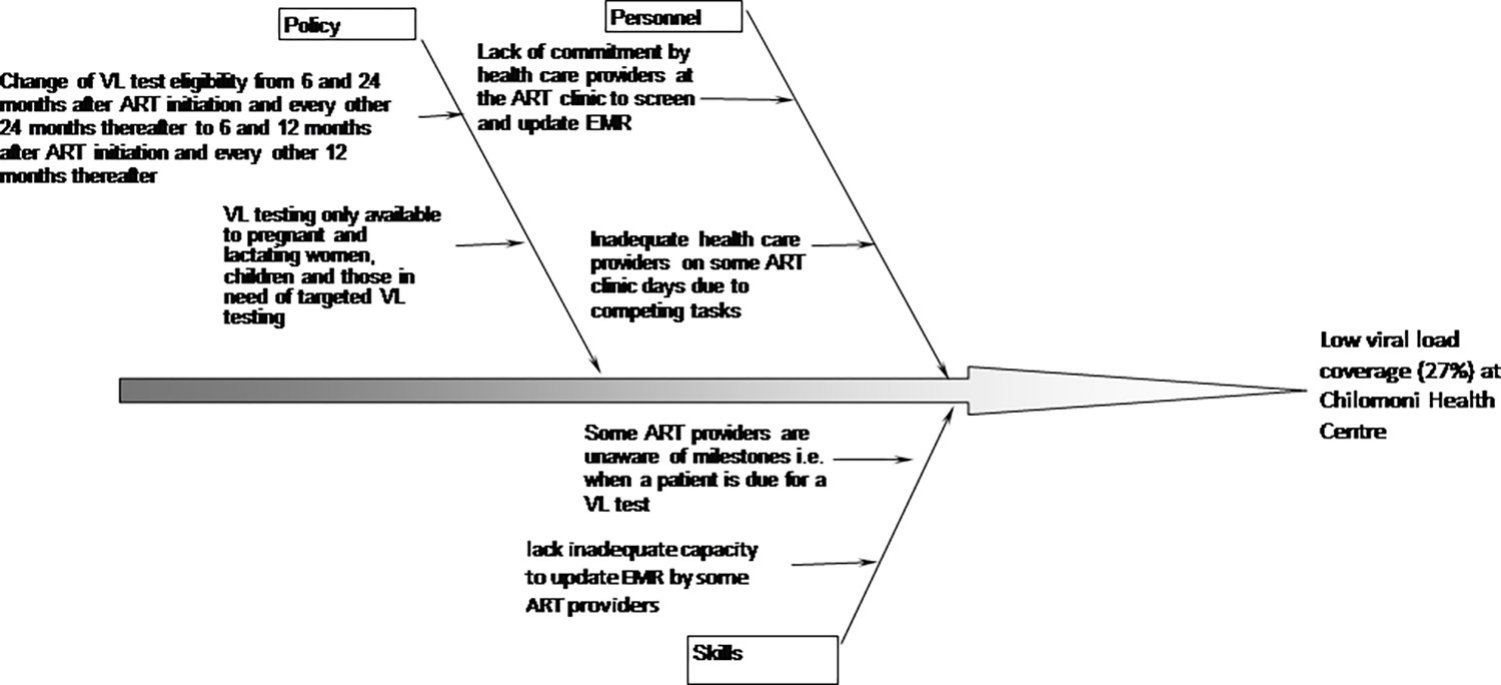
Fishbone diagram of root causes of low VL testing coverage at Chilomoni health centre, Blantyre.

*Step 2*: *Root-cause analysis and prioritization*. A root cause analysis of factors that have led to low VL coverage at Chilomoni health centre were established using a fishbone diagram. A list of guiding questions was developed to ensure an exhaustive analysis, including the use of the 5 ‘Why’ approach (asking ‘Why’ 5 times) for each mentioned factor **(Fig 1**).

**Fig 2:**
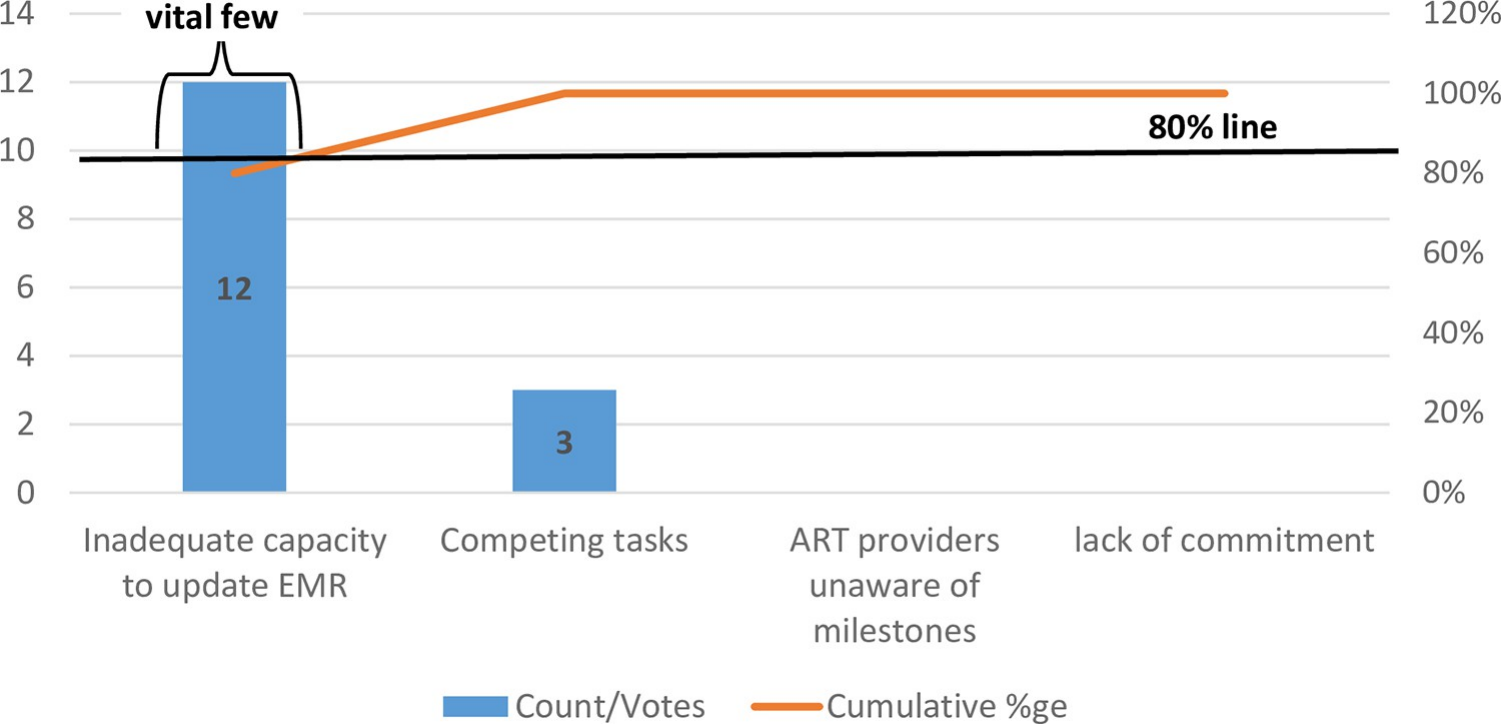
Pareto chart of prioritizing factors affecting low VL coverage at Chilomoni health centre, Blantyre.

Factors that affect VL testing coverage at the health facility were prioritized using a pareto chart **(Fig 2).** This allowed for the easy determination of factors with the highest priority and to guide the development of change ideas. Pareto charts are based on the principle that 80% of the problem is because of 20% of the possible causes, the vital few. Factors under ‘Policy’ from the fishbone diagram were not included in the pareto chart because they were not within the control of the health care providers at the health facility.

Health care providers voted for factors they perceived highly contributed to the problem of low VL coverage. Updating EMR records was identified as the problem with the highest priority followed by competing tasks that caused inadequate screening.

**Fig 3:**
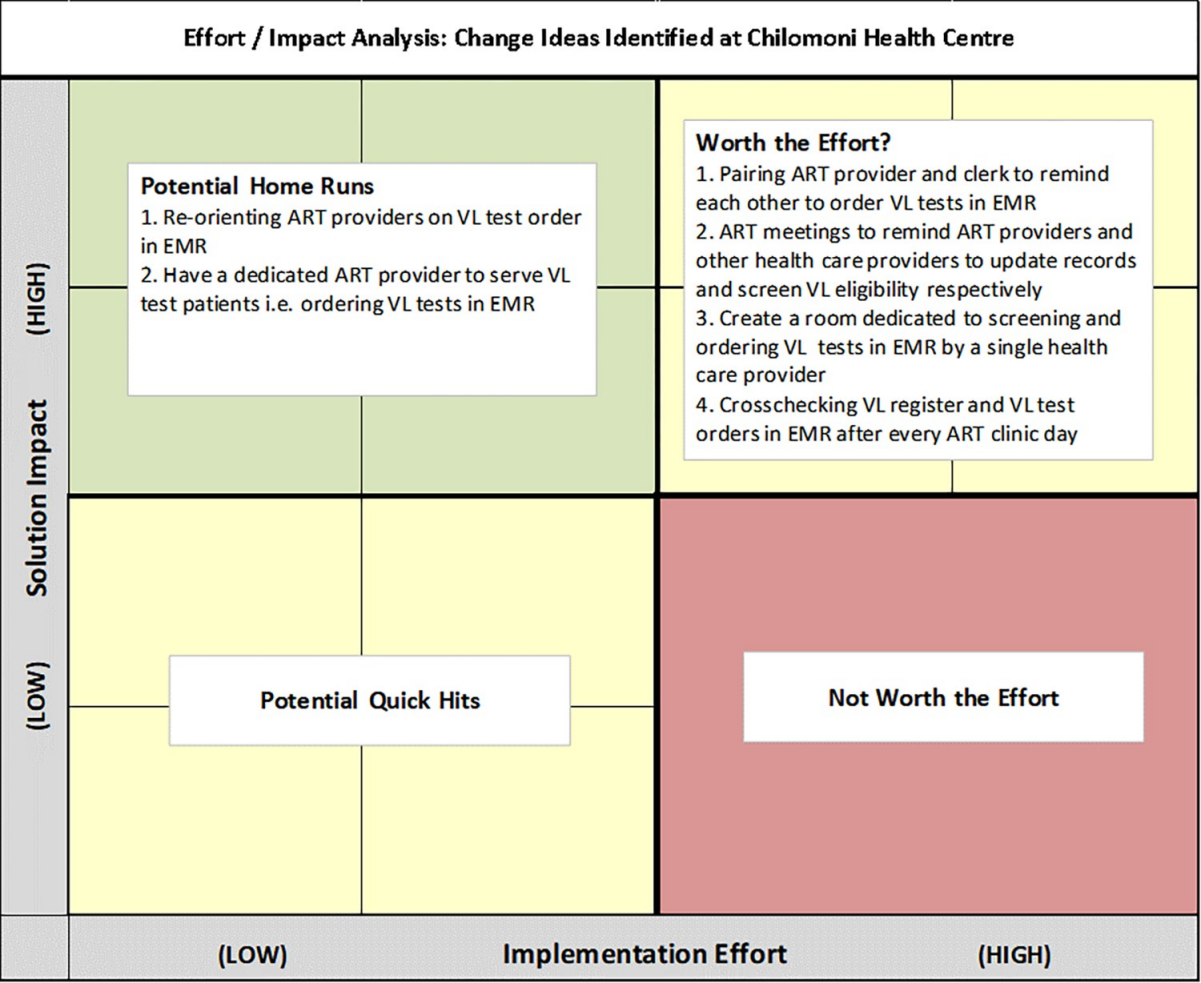
Effort-Impact Matrix of change ideas identified at Chilomoni health centre in Blantyre.

*Step 3*: *Developing a change idea and prioritization*. Possible change ideas that could be implemented were identified by health care providers in a consultative brainstorming session. During the session, health care providers were encouraged to identify change ideas that can be implemented even after control of the COVID-19 situation and delivery of HIV services is back to normal. Health care providers suggested 6 change ideas that could be implemented. Change ideas were prioritized on an effort-impact matrix in order to determine which change idea to implement **(Fig 3)**. The matrix prioritizes change idea(s) based on the effort needed to implement as well as the impact that is cultivated from implementing the change idea. The change ideas requiring the least effort but with high impact were implemented.

#### Phase II

*Step 4*: *Implementation and testing of change ideas (intervention period)*. Emanating from the effort-impact matrix, two (2) change ideas were implemented and tested using Plan-Do-Study-Act (PDSA) cycles embedded in the MFI framework (**Table 1**). The PDSA cycle provides a series of steps for planning, executing, analysing and making decisions on a tested change idea. Change ideas were tested in 5 PDSA cycles and with each cycle lasting one week. During each PDSA cycle data on the outcome measure, VL testing coverage, was collected using paper-based forms that were entered in MS Excel. Data on process and balancing measures were also documented.

After each PDSA cycle, the outcome measure and all other related measures were reviewed with the QI team representatives and key health care providers who were present at the ART clinic during the testing of the previous change idea. Key health care providers included ART providers (nurses and clinician), Expert Clients working at the reception, HSAs working in the VL and EID rooms and clerks. It is at these reviews that a decision was made on the previously tested change idea and the next PDSA cycle was planned.

#### Description of PDSA cycles

*PDSA cycle 1*: *Re-orienting ART providers on VL test order in EMR*. The VL focal and data clerk reoriented ART providers present on that ART clinic day on how to order a VL test in EMR 15 minutes prior to start of the ART clinic.

*PDSA cycle 2*: *Re-orienting ART providers on VL test order in EMR adapted*. The same change idea from the first PDSA cycle was implemented. However, the VL focal appointed a team leader who was responsible for assigning responsibilities to health care providers present at the ART clinic, including screening for VL eligibility. A different set of health care providers worked on each ART clinic day.

*PDSA cycle 3*: *Dedicated ART provider to serve VL tested patients*. One ART provider was responsible for serving patients who had received a VL test. The designated ART provider sat in the office nearest to the VL test room on that clinic day. All patients including MIPs were asked to go through the reception for screening before accessing any service at the ART clinic, including EID services. Health passports of eligible patients were held at the reception and forwarded to the VL testing room. After the VL test, health passports and patients were forwarded to the dedicated ART providers’ office. Receipt of VL test was documented in the patients’ health passport.

*PDSA cycle 4*: *Dedicated ART provider to serve VL tested patients adapted*. The same change idea from PDSA 3 was adapted in PDSA 4 to include a VL screening and VL testing roster. Predetermination of health care providers responsible for VL eligibility screening and collecting samples for VL testing was done to promote planning and ensure these core components had dedicated people who knew and had prepared for their task on that ART clinic day.

*PDSA cycle 5*: *Dedicated ART provider to serve VL tested patients adapted*. The change idea in PDSA 4 was modified in PDSA 5 to include a hardcover that was used by the health care provider responsible for screening to document ART numbers of screened patients and last VL test dates. This was particularly for those patients whose previous ART printed sticker did not show VL test results. This change idea was eventually adopted for use.

#### Step 5: Monitoring of performance of outcome measure after PDSA cycles (post-intervention period)

After a change idea was adopted as the new way of operation, PDSA cycles were stopped and performance monitoring of the VL testing coverage continued. This was done to determine if continued implementation of the adopted change idea would bring about the same changes and whether the change idea was sustainable. Monitoring performance of the outcome measure after PDSA cycles lasted 5 weeks and no weekly reviews were done during this period. However, a presentation on the entire QI project was made at the health facility, including the monitoring period data.

### Data analysis, documentation and dissemination

Data on individuals due for a VL test regardless of whether they got a VL test or not was imported from Microsoft Excel to STATA for descriptive statistics.

Run charts and statistical process control (SPC) methods particularly Proportion charts (P-charts), were also used to analyse and review the performance of the outcome measure on a weekly basis throughout the study period.

### Ethical consideration

Prior to the start of the study, administrative permission was obtained from the Blantyre District Health Office. Ethical clearance was also obtained from the Human Research Ethics Committee (HREC) of University of the Witwatersrand (Protocol No.: M1911158) in South Africa and from the National Health Sciences Research Committee (NHSRC) in Malawi (Protocol No.: 20/01/2455).

Written consent was obtained from health care providers and patients whose records were used for the prospective record review. Names and identifiable data of patients were not documented and data was password protected.

## Results

Overall, 428 records belonging to 370 pregnant and lacting women as well as children were reviewed during the study from baseline to the end. Some patients visited the ART clinic twice during the study; at baseline and during the intervention or post-intervention period hence they had more records than other patients. However, each visit was treated as a unique record during weekly tabulations of VL testing coverage.

Generally, more individuals came to the ART clinic at baseline (55%) than during and after PDSA ccycles ***(Table 2)***. In addition, there were fewer children (32%) than pregnant and lactating women that came through to the ART clinic.

**Table 2:** Descriptive characteristics of study participants and VL testing data.

Variables	At Baseline [n = 203 (55%)]	During PDSA cycles [n = 94 (25%)]	After PDSA cycles (post-intervention) [n = 73(20%)]	Overall [n = 370 (100%)]
**Pregnant and lactating women**	**139 (68%)**	**67 (71%)**	**55 (75%)**	**261 (71%)**
**Children**	**64 (32%)**	**27 (29%)**	**18 (25%)**	**109 (29%)**
***Male***	37 (58%)	19 (70%)	11(61%)	67 (61%)
***Female***	27 (42%)	8 (30%)	7 (39%)	42 (39%)
***0–14 years***	54 (84%)	21 (78%)	15 (83%)	90 (83%)
***15–17 years***	10 (16%)	6 (22%)	3 (17%)	19 (17%)
**VL tested**	**69 (34%)**	**62 (66%)**	**61 (84%)**	**192 (52%)**
***Children 0–14 years***	8(15%)	10 (47%)	13(87%)	31 (34%)
***Children 15–17 years***	2 (20%)	6 (100%)	3(100%)	11 (58%)
***Pregnant and lactating women***	59 (43%)	46 (66%)	45(82%)	150 (57%)

Overall, VL testing coverage improved from 27% to 71% by the end of the study. Based on the period in the study at which each individual received a VL test, a large proportion received their VL test during the monitoring period (84%) than at baseline (34%) and during PDSA cycles (66%). Among children aged 0–14 years, only 15% had their VL tested during baseline. During PDSA cycles, there was an increase to 47% and further increase during the monitoring phase, 87%. For children aged between 15 and 17, all individuals in this age bracket got their VL tested in the PDSA cycle phase and monitoring phase. There was also an increase in the adults as those who got tested increased from 43% at baseline to 66% during testing of change ideas, and to 82% after PDSA cycles.

**Fig 4:**
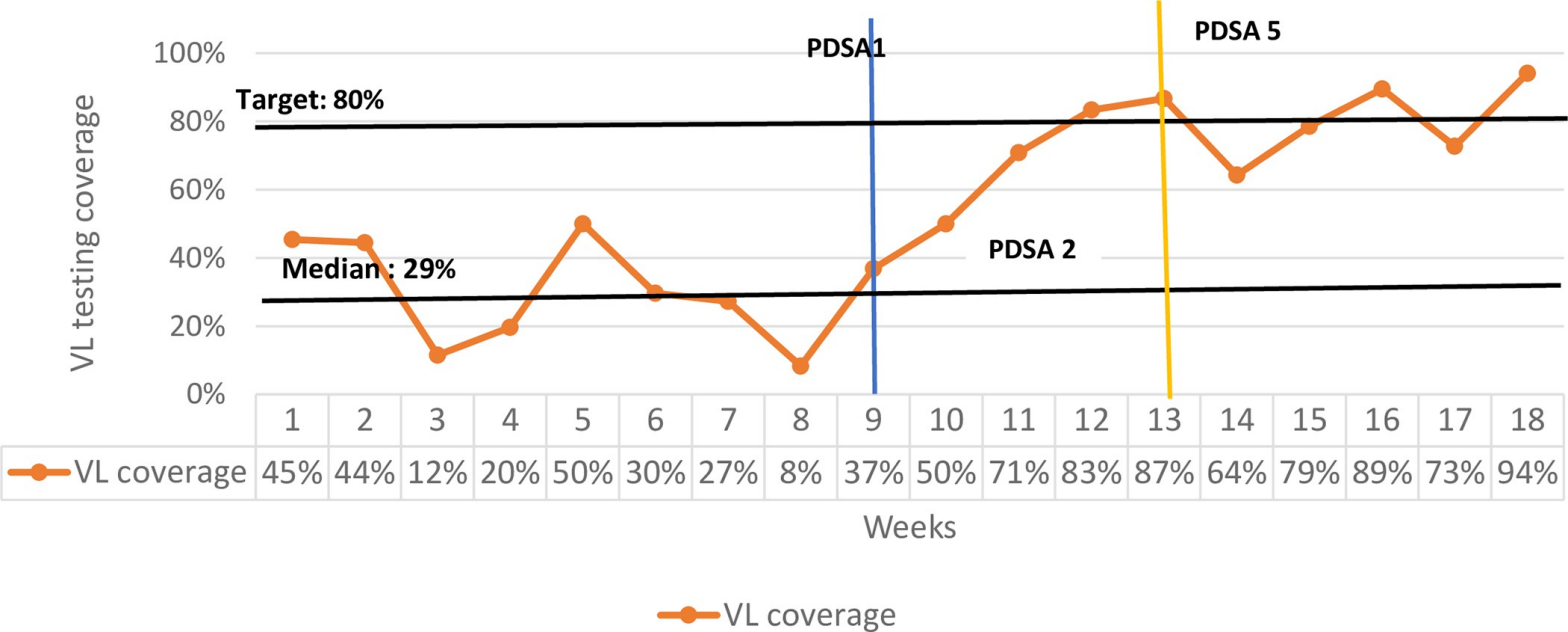
Run chart of VL testing coverage at Chilomoni health center in Blantyre.

Weekly VL testing coverage measurements during the baseline period ranged from 8% to 50%, with week 8 having the lowest coverage and week 5 the highest (**Fig 4**).

Inception of PDSA cycles started at week 9. From week 9 to week 13, there was an upward trend with implementation of each PDSA cycle. The adapted change idea during week 10 was implemented because it was noted that apart from inadequate capacity to make VL test orders in EMR, screening for VL test eligibility was also inadequate. However, the change idea implemented in PDSA cycle 2 (week 10) was abandoned all together due to low proportions of VL tests made in the EMR, 50% and 29% of the VL tests ordered in EMR were incorrect. This was particularly so for individuals who were missed during screening at the reception and were discovered in the ART providers office. The ART provider would order a VL test in EMR, give medication to the patient and sent them to the VL room. This resulted in patients skipping the sample collection process for a VL test.

At week 11 (PDSA cycle 3), a new change idea was implemented and emphasis was on intensified screening at the ART clinic reception, rather than the waiting area. There were no incorrect lab orders made in EMR in this PDSA cycle and 95% of the VL tests conducted were ordered in EMR. The health facility was able to hit the 80% target at weeks 12 and 13 (PDSA cycles 4 and 5) and all VL tests conducted were ordered in EMR, with no incorrect orders being made in the system. At PDSA 4, an adaptation of the change idea in PDSA 3 was implemented. This was done after noticing that identifying people responsible for screening and and collecting samples for VL testing on the same day was a challenge and delayed service delivery. An adapation of PDSA 4 was implemented during PDSA 5 after establishing that some patient records in the EMR were not up to date, and yet the patient had recieved a VL test. The *ART provider dedicated to serve VL tested patients* change idea was adopted after PDSA 5 and was implemented throughout the monitoring phase.

Monitoring of the outcome after adopting the change idea started at week 14. Coverage of VL testing dropped to 60% at week 14 and went up to 90% by week 16. However, VL coverage dropped again at week 17 to 71% and then finally went up again to 94% in the final week.

**Fig 5:**
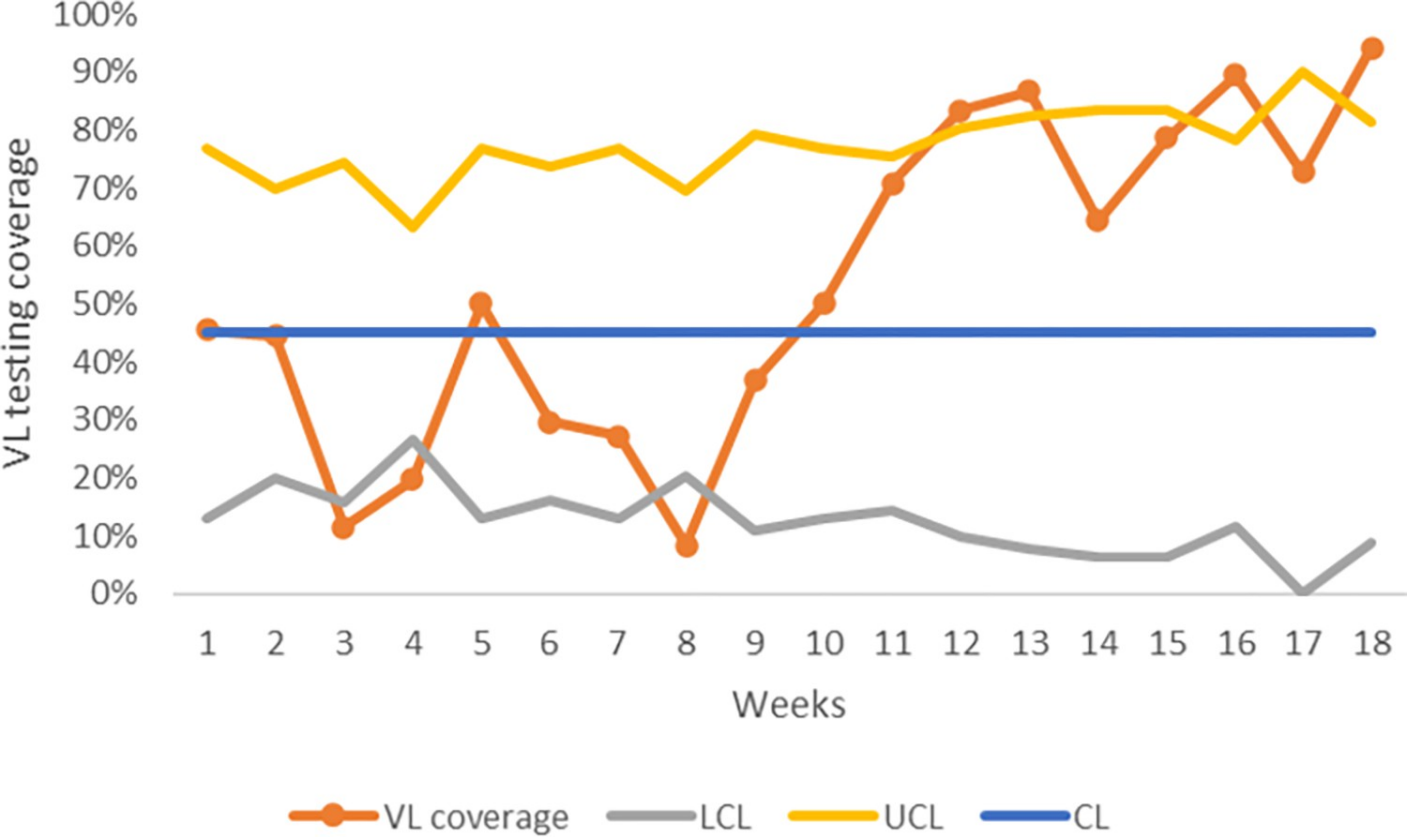
P-Chart of VL testing coverage at Chilomoni health center with upper and lower control limit.

Viral load testing coverage during baseline (weeks 3, 4 and 8) and during the intervention and post intervention period (weeks 12, 13, 16 and 18) show special cause variation; VL testing coverage was below the lower control limit (LCL) and above the upper control limit (UCL) (**Fig 5**).

Reasons for special cause variation during baseline coud not identified due to restrospective data collection. However, during the intervention period particularly at weeks 12 and13, special cause variations were due to the change idea that was being implemented. Special cause variations in a process can be caused by the intervention being implemented [16]. The change idea implemented at weeks 12 and 13 was adopted and the same effect was noticed during the post intervention period at weeks 16 and 18.

## Discussion

Our study set out to improve VL testing coverage at Chilomoni health centre using a QI approach, particularly using the MFI. Re-orientation of ART providers on how to order VL tests in EMR and making some changes in the VL testing process brought about improvement in VL testing coverage. Alterations in the VL testing process included changing the point of screening for VL eligibility from the waiting area to the reception while documenting ART numbers of screened clients, and escorting patients to the VL testing room. On the other hand, shortage of staff caused fluctuations in VL testing coverage particularly during the post intervention period. Shortage of staff was identified as the utmost facility-specific barrier to VL monitoring in another study conducted in Malawi [17]. The situation in our study was further exacerbated by the report of the first COVID-19 case at the health facility during the last PDSA cycle which resulted in immediate quarantine of some health care providers from the ART clinic hence a reduction in staff. This resulted in a drop in VL testing coverage at week 14. Viral load testing coverage also dropped at week 17 due to another episode of inadequate staff; some health care providers were attending a TB training, others were supporting the COVID-19 quarantine centre in the district and some were still in quarantine.

Our study findings are aligned with other study results that have demonstrated that QI interventions can empower health care providers to identify problems within their work enviroment and apply necessary improvements depending on context [18]. Using QI approaches assisted 10 health facilities in Sierra Leone to improve Provider Initiated HIV Testing and Counselling (PITC) from 4% to 95% [18]. In South Africa, VL testing coverage for patients with recognised treatment failure improved from 63% to 94% using a VL champion through a QI study in 3 clinics based in Kwazulu Natal’s eThikwini health district [19].

Similar to the baseline VL testing coverage in our study, Blantyre district’s VL testing coverage was reported to be between 25% and 30% in 2019 [14]. Factors affecting VL testing coverage established in this study; in adequate capacity by ART providers to update EMRs, competing tasks, ART providers being unaware of milestones and lack of commitment, also agree with other study findings conducted in other limited resource countries, including Malawi. For instance, inadequate documention of samples collected for VL tests, clinical knowledge gaps and shortage of staff that led to overwhelming responsibilities were identified as barriers to VL testing in Malawi [19, 20]. In our study, the most prominent factors were inadequate capacity by ART providers to update EMRs and competing tasks. As such, all change ideas implemented from the second PDSA to the last cycle aimed at addressing these factors as a package.

Emphasis in the first implemented change idea was reorienting ART providers, an informal method of training, and screening of VL test eligibility. Training of health care providers has the ability to improve willingness to use Electronic Health Records (EHR) systems as well as effective use of the system, including using advanced features in EHR like order tests [21]. However, in our study training did not bring about substantial change as would have been anticipated. This can be attributed to the informal nature of the orientation itself but also time taken to conduct the exercise. The second change idea was more complex. Deciding to have the reception as the starting point for all patients visiting the ART clinic made screening efficient and identification of VL eligible patients easier. In addition, escorting patients eligible for VL testing to the VL room while holding on to their health passports ensured that the patients made it to the VL room, a sample for VL testing was collected and documented in their health passport. Accompanying HIV positive pregnant mothers with low CD4 count by ANC clinic staff to the Highly Active Antiretroviral Therapy (HAART) clinic led to improved access to HAART in ANC clinics from 10% to 25% in primary care sites in Cape Metro Subdistrict, South Africa [22]. A dedicated ART provider situated in a room next to the VL room also enabled the ART provider to remember to update VL test orders in EMR hence all VL tests were reflected in the system.

Contexts in which structures and guidelines for QI are already existent provide a conducive environment for implementing QI projects [18]. The existence of a QI team and knowledge of QI at the health facility was an important enabler in this project. Other facilitators in our study included committed health care providers to the QI project, good data collection and extraction of records and local leadership buy-in. Good communication and review of VL testing coverage with relevant health care providers was also another facilitator. On the other hand, COVID-19 was a barrier as it slowed down processes and affected the study population. A reliable data system at the health facility was also another barrier although this was dealt with through a catchup data entry exercise of all VL testing data. The existence of a reliable data system that is functional, up to date and allows easy extraction of data can not be emphasized enough if a QI intervention of this nature is to succeed as this is the source of all the evidence of whether progress is being made or not.

One of the limitations of our study was the lack of a comparison site to be able to evaluate the difference in VL testing coverage. In addition, the study duration was short. As a result, there was inadequate time to assess sustainability of improved VL testing coverage due to the adopted and implemented change idea. External validity of the change ideas implemented in this study is uncertain as these were specific to Chilomoni health center and its problems. Nevertheless, other health facilities are encouraged to employ the methods and components of interventions that apply to their setting. On a related note, our study expected to have a larger sample size than what was realized. Sample size was affected by change in HIV service delivery guidelines because of COVID-19; VL testing was only available to certain groups of individuals. On the other hand, strengths of the study included using local data and expert opinion as well as available resources and structures. There was no creation of parallel structures just to cater for the QI study. This helped to promote a sense of ownership and commitment from health care providers. It also assisted in minimizing costs that come with introducing new structures in a system [23]. Use of the MFI as a guiding framework will also allow comparison of our study methodology and results with other studies.

## Conclusion and recommendations

Our study experience demonstrates that VL testing coverage can be improved using a QI approach, particularly using the MFI framework. However, there is need for investment by government through the Ministry of Health in reliable and efficient data systems to enable health care providers easily access data for monitoring and QI interventions. Investment in data systems would allow health care providers to be able to easily extract data and monitor progress without having to go through the tedious process of reviewing all individual patient records just to determine VL test eligibility for instance. The appointment calendar in EMR provides a list of names of individuals coming to the ART clinic on any particular date, including their age, sex and date of ART initiation. We suggest including VL test eligibility data on this appointment list in EMR to improve screening and data extraction processes hence improving VL testing coverage. Continued success and improvement will be dependent on the QI leadership as well as commitment of health care providers.

Further research is needed to evaluate sustainability of the improved VL testing coverage at the health facility, including patient related factors that affect VL testing coverage with a focus on the paediatric population due to low VL testing coverage observed during the study at baseline. It would also be vital to assess the impact of change in policy from VL testing every two years after the first initial VL test to annually, including the new guidelines for VL testing amid COVID-19.

## Supporting information

S1 Dataset (XLS)
